# Editorial: Women in antimicrobials, resistance and chemotherapy: 2022

**DOI:** 10.3389/fmicb.2023.1148777

**Published:** 2023-02-08

**Authors:** Ana R. Freitas, Didem Şen Karaman, Cindy Shuan Ju Teh

**Affiliations:** ^1^TOXRUN—Toxicology Research Unit, University Institute of Health Sciences, CESPU, CRL, Gandra, Portugal; ^2^UCIBIO/REQUIMTE, Applied Molecular Biosciences Unit, Department of Biological Sciences, Laboratory of Microbiology, Faculty of Pharmacy, University of Porto, Porto, Portugal; ^3^Associate Laboratory i4HB, Institute for Health and Bioeconomy, Faculty of Pharmacy, University of Porto, Porto, Portugal; ^4^Department of Biomedical Engineering, Faculty of Engineering and Architecture, Izmir Katip Çelebi University, Izmir, Türkiye; ^5^Department of Medical Microbiology, Faculty of Medicine, Universiti Malaya, Kuala Lumpur, Malaysia

**Keywords:** Antimicrobial Resistance (AMR), women's empowerment, biocides, co-selection, genomics, One Health

In celebration of International Women's Day 2022, Frontiers in Microbiology launched this “*Women in antimicrobials, resistance, and chemotherapy: 2022*” Research Topic, a dedicated Frontiers Research Topic totally handled by women as editors aimed at celebrating the achievements of women in this field. The contribution of many women to the history of STEM (science, technology, engineering, and mathematics) is undeniable, with a few remarkable examples throughout time including Ada Lovelace (arguably) considered the first computer programmer in the middle 1880's, Rosalind Franklin discovering the structure of DNA in the 1950's, Barbara McClintock for the discovery of jumping genes in 1983 or Jennifer Doudna together with Emmanuelle Charpentier for their 2012 discovery that a bacterial immune system called CRISPR can be repurposed to edit DNA (Rigby, 2021). Who does not know the physicist and chemist Marie Curie as the first female recipient of a Nobel Prize for Physics in 1903 (together with her husband) and then alone for Chemistry in 1911, in recognition of her work in radioactivity?

But women's dedication did not always gain the recognition they may deserve over the years, with sexism, racism, and single-use of conventional sources, among other factors, contributing to the undervaluation of their achievements (Editorial: Women must not be obscured in science's history, 2021). In fact, even though women scientists are leading groundbreaking work in different science areas worldwide, they still represent only ~30% of researchers globally and <4% of Nobel Prizes for science have ever been awarded to women (UNESCO, 2015).

Gender equality and the empowerment of all women and girls is one of the goals under the 2030 Agenda with obvious and broad benefits for sustainable development across different sectors and society (UN Women, 2018). The same occurs with Antimicrobial Resistance (AMR) which is necessary to ensure effective and equitable impact. For that, there is a need to understand how men and women may be different at risk of or impacted by AMR, and how this interconnects with other issues such as income and education, occupation, or geographic location [several examples are provided in World Health Organization (2018)]. While there is still a lot to be done to achieve true gender equality in science, the papers published on this Research Topic (five original Research Articles) highlight the strength and up-to-date research about Antimicrobial Resistance performed by female researchers across distant areas including Argentina, China, Europe, India, New Zealand, and the USA.

Souza et al. analyzed the phylogenetic distribution of genes conferring resistance to heavy metals, biocides, and antimicrobial compounds in 394 genome sequences of clinical human-derived *S. enterica* (78 STs, 68 serotypes) obtained from New Hampshire, USA during 2017–2020. Different operons related to heavy metal resistance (arsenic, copper, gold, mercury, silver, and tellurite) and genes associated with resistance to quaternary ammonium compounds (*qacEdelta1, qacL*, and *sugE1*) were differentially distributed across multiple STs and serotypes, with the gold operon (*gesABC-golTSB*) being predominantly present in 99.2% of the genomes. A strong negative or purifying selection able to remove deleterious mutations was demonstrated for these genes, with some frequently co-occurring with AMR genes within common plasmid types. Given the wide distribution and routine use of different heavy metals and biocides in household and industrial products, this study reinforces the need for continued surveillance of strains that frequently carry such adaptive traits able to act as agents of co-selection of AMR genes.

Collis et al. reported the prevalence and distribution of extended-spectrum β-lactamase (ESBL)- and AmpC-producing *Escherichia coli* from farm environmental samples collected over a 15-month period in two New Zealand dairy farms that differed in size, number of cows and effluent management strategies during the study period. Overall, 52 *E. coli* strains were isolated and 33 were classified as multidrug-resistant (MDR). All the strains were resistant to cefpodoxime, followed by 89% to cefoxitin, 79% to streptomycin, 65% to cefotaxime, and 64% to tetracycline. Of the 52 strains, 46 and 32 strains were identified as AmpC- and ESBL-producer, respectively. The ESBL-producers were only isolated from the larger farm while AmpC-producers were detected in both farms (except the plasmid-mediated *bla*CMY−2 gene predominantly detected in the larger farm). The predominant *E. coli* phylogroups were B1 (35%) and C (33%). The subset of AmpC- and ESBL-producing *E. coli* sequenced in this study showed a diverse molecular relationship with eight sequence types identified. Overall, the isolation of the ESBL- and AmpC-producing *E. coli* was not associated with periods of elevated antimicrobial usage in the farms.

Huang et al. determined the distribution and AMR phenotypes of 257 bacterial isolates from the intestine of 115 red swamp crayfish samples, which are one of the most popular and commercially farmed aquatic products in China. Most isolates corresponded to *Citrobacter* sp. and *Aeromonas* sp., with the majority being susceptible to all tested antibiotics. *Pseudomonas sp*., *Aeromonas caviae*, and *E. coli* were multidrug-resistant, with the last being identified as ST48 and carrying the carbapenemases gene *bla*_NDM − 5_, which is accepted as a last-line Antimicrobial Resistance gene. Because of the high potential for transmission to humans through the food chain, red swam crayfish has been pointed out for acting as a reservoir of last-line antibiotic resistance genes posing a serious public health risk.

Cañada-García et al. analyzed 50 imipenemase-producing Enterobacterales (IMP-Ent) circulating in 19 Spanish hospitals over 9 years (between January 2012–December 2021) by deeply characterizing their population structure and resistomes, and evaluating different phenotypic methods for carbapenemase detection. Although still generally infrequent, their findings revealed that IMP-8-producing *Klebsiella pneumoniae* and IMP-22- producing *Enterobacter roggenkampii* constitute the most frequent IMP-producing *Enterobacterales* nationwide, that colistin and amikacin were the most active non-carbapenem antibiotics and, importantly, that inhibition with EDTA or dipicolinic acid presented false negative results in some IMP-producing strains. As they have already been linked to nosocomial outbreaks, active surveillance is continuously warranted to limit IMP-Ent dissemination.

Sharma et al. developed an automated pipeline, Galaxy-ASIST, for the characterization of clinical isolates. Breakpoint mapping and phenotypic classification can be performed with the input of antimicrobial susceptibility profiles, while AMR determinants can be mapped based on whole genome sequencing data. This platform is able to reduce the complexity of data analysis and offers a user-friendly interface for mapping global guidelines for reporting antimicrobial susceptibility profiles, allowing non-bioinformatics researchers to perform analysis without the need for any coding skills. The current study focused on *Acinetobacter baumannii* but can be scaled for all priority pathogens in the future. This platform is freely available at: https://ab-openlab.csir.res.in/asist.

Taken together, these studies illustrate the high quality of studies headed by diverse women scientists addressing one of the top 10 global public health threats of our times, which is Antimicrobial Resistance. We hope that these articles can engage readers working in the field of AMR to explore and tackle AMR from multi-disciplinary Public Health perspectives.

## Author contributions

All authors have contributed to this Editorial and approved it for publication.
